# Supplementary material for: Comparison of generic drug prices in Korea and eight high-income countries across four therapeutic classes
Source: PLoS One. 2025 Mar 11;20(3):e0319674. doi: 10.1371/journal.pone.0319674 (PMC11896052; doi:10.1371/journal.pone.0319674)
Supplement: Supplementary Table 1 — (DOCX) [file pone.0319674.s001.docx]

Supplementary Table 1. Study drugs

| Drug class | ATC5 code | ATC level name | Strength |
| --- | --- | --- | --- |
| Antidiabetic drug | A10BA02 | metformin | 250mg |
|  | A10BA02 | metformin | 500mg |
|  | A10BA02 | metformin | 500mg |
|  | A10BA02 | metformin | 850mg |
|  | A10BA02 | metformin | 1g |
|  | A10BA02 | metformin | 750mg |
|  | A10BB01 | glibenclamide | 5mg |
|  | A10BB09 | gliclazide | 80mg |
|  | A10BB09 | gliclazide | 30mg |
|  | A10BB12 | glimepiride | 1mg |
|  | A10BB12 | glimepiride | 2mg |
|  | A10BB12 | glimepiride | 3mg |
|  | A10BB12 | glimepiride | 4mg |
|  | A10BD02 | metformin and sulfonylureas | 2.5mg+500mg |
|  | A10BD02 | metformin and sulfonylureas | 5mg+500mg |
|  | A10BD02 | metformin and sulfonylureas | 1.25mg+250mg |
|  | A10BD05 | metformin and pioglitazone | 850mg+15mg |
|  | A10BD06 | glimepiride and pioglitazone | 2mg+30mg |
|  | A10BD06 | glimepiride and pioglitazone | 4mg+30mg |
|  | A10BD13 | metformin and alogliptin | 12.5mg+1g |
|  | A10BF01 | acarbose | 100mg |
|  | A10BF01 | acarbose | 50mg |
|  | A10BF02 | miglitol | 50mg |
|  | A10BF02 | miglitol | 100mg |
|  | A10BF03 | voglibose | 200μg |
|  | A10BF03 | voglibose | 200μg |
|  | A10BF03 | voglibose | 300μg |
|  | A10BF03 | voglibose | 300Y |
|  | A10BG03 | pioglitazone | 15mg |
|  | A10BG03 | pioglitazone | 30mg |
|  | A10BH01 | sitagliptin | 25mg |
|  | A10BH01 | sitagliptin | 50mg |
|  | A10BH01 | sitagliptin | 100mg |
|  | A10BH02 | vildagliptin | 50mg |
|  | A10BH03 | saxagliptin | 2.5mg |
|  | A10BH03 | saxagliptin | 5mg |
|  | A10BH04 | alogliptin | 12.5mg |
|  | A10BH04 | alogliptin | 25mg |
|  | A10BX02 | repaglinide | 1mg |
|  | A10BX02 | repaglinide | 2mg |
|  | A10BX03 | nateglinide | 90mg |
|  | A10BX03 | nateglinide | 120mg |
| Lipid-modifying agents | C10AA01 | simvastatin | 20mg |
|  | C10AA01 | simvastatin | 40mg |
|  | C10AA01 | simvastatin | 10mg |
|  | C10AA02 | lovastatin | 20mg |
|  | C10AA03 | pravastatin | 10mg |
|  | C10AA03 | pravastatin | 5mg |
|  | C10AA03 | pravastatin | 20mg |
|  | C10AA03 | pravastatin | 40mg |
|  | C10AA04 | fluvastatin | 20mg |
|  | C10AA04 | fluvastatin | 40mg |
|  | C10AA04 | fluvastatin | 80mg |
|  | C10AA05 | atorvastatin | 10mg |
|  | C10AA05 | atorvastatin | 20mg |
|  | C10AA05 | atorvastatin | 40mg |
|  | C10AA05 | atorvastatin | 80mg |
|  | C10AA07 | rosuvastatin | 10mg |
|  | C10AA07 | rosuvastatin | 20mg |
|  | C10AA07 | rosuvastatin | 5mg |
|  | C10AA07 | rosuvastatin | 2.5mg |
|  | C10AA08 | pitavastatin | 2mg |
|  | C10AA08 | pitavastatin | 1mg |
|  | C10AA08 | pitavastatin | 4mg |
|  | C10AB04 | gemfibrozil | 300mg |
|  | C10AB05 | fenofibrate | 250mg |
|  | C10AB05 | fenofibrate | 200mg |
|  | C10AB05 | fenofibrate | 160mg |
|  | C10AB05 | fenofibrate | 160mg |
|  | C10AB11 | choline fenofibrate | 135mg |
|  | C10AX06 | omega-3-triglycerides incl. other esters and acids | 2g |
|  | C10AX06 | omega-3-triglycerides incl. other esters and acids | 1g |
|  | C10AX09 | ezetimibe | 10mg |
|  | C10BA02 | simvastatin and ezetimibe | 10mg+10mg |
|  | C10BA02 | simvastatin and ezetimibe | 10mg+20mg |
|  | C10BA02 | simvastatin and ezetimibe | 10mg+40mg |
|  | C10BA05 | atorvastatin and ezetimibe | 10mg+10mg |
|  | C10BA05 | atorvastatin and ezetimibe | 20mg+10mg |
|  | C10BA05 | atorvastatin and ezetimibe | 40mg+10mg |
|  | C10BA06 | rosuvastatin and ezetimibe | 10mg+5mg |
|  | C10BA06 | rosuvastatin and ezetimibe | 10mg+10mg |
|  | C10BA06 | rosuvastatin and ezetimibe | 10mg+20mg |
| Anti-hypertensive drugs | C02CA04 | doxazosin | 1mg |
|  | C02CA04 | doxazosin | 2mg |
|  | C03BA08 | metolazone | 5mg |
|  | C03BA11 | indapamide | 1.5mg |
|  | C03BA11 | indapamide | 2.5mg |
|  | C03CA01 | furosemide | 40mg |
|  | C03CA04 | torasemide | 10mg |
|  | C03CA04 | torasemide | 2.5mg |
|  | C03CA04 | torasemide | 5mg |
|  | C03DA01 | spironolactone | 25mg |
|  | C03DA01 | spironolactone | 50mg |
|  | C03EA01 | hydrochlorothiazide and potassium-sparing agents | 25mg+25mg |
|  | C07AA05 | propranolol | 10mg |
|  | C07AA05 | propranolol | 40mg |
|  | C07AA12 | nadolol | 40mg |
|  | C07AB03 | atenolol | 25mg |
|  | C07AB03 | atenolol | 50mg |
|  | C07AB05 | betaxolol | 10mg |
|  | C07AB05 | betaxolol | 20mg |
|  | C07AB07 | bisoprolol | 10mg |
|  | C07AB07 | bisoprolol | 5mg |
|  | C07AB07 | bisoprolol | 2.5mg |
|  | C07AB08 | celiprolol | 200mg |
|  | C07AB09 | esmolol | 2.5g |
|  | C07AB12 | nebivolol | 5mg |
|  | C07AB12 | nebivolol | 2.5mg |
|  | C07AB12 | nebivolol | 1.25mg |
|  | C07AG01 | labetalol | 100mg |
|  | C07AG02 | carvedilol | 12.5mg |
|  | C07AG02 | carvedilol | 25mg |
|  | C07AG02 | carvedilol | 6.25mg |
|  | C07BB07 | bisoprolol and thiazides | 2.5mg+6.25mg |
|  | C07BB07 | bisoprolol and thiazides | 5mg+6.25mg |
|  | C07BB07 | bisoprolol and thiazides | 10mg+6.25mg |
|  | C07CB03 | atenolol and other diuretics | 50mg+12.5mg |
|  | C08CA01 | amlodipine | 10mg |
|  | C08CA01 | amlodipine | 2.5mg |
|  | C08CA01 | amlodipine | 5mg |
|  | C08CA02 | felodipine | 5mg |
|  | C08CA02 | felodipine | 2.5mg |
|  | C08CA03 | isradipine | 5mg |
|  | C08CA04 | nicardipine | 10mg |
|  | C08CA04 | nicardipine | 20mg |
|  | C08CA05 | nifedipine | 10mg |
|  | C08CA05 | nifedipine | 5mg |
|  | C08CA05 | nifedipine | 40mg |
|  | C08CA09 | lacidipine | 2mg |
|  | C08CA09 | lacidipine | 4mg |
|  | C08CA09 | lacidipine | 6mg |
|  | C08CA13 | lercanidipine | 10mg |
|  | C08CA14 | cilnidipine | 10mg |
|  | C08CA15 | benidipine | 2mg |
|  | C08CA15 | benidipine | 4mg |
|  | C08CA15 | benidipine | 8mg |
|  | C08CA17 | levamlodipine | 2.5mg |
|  | C08CA17 | levamlodipine | 5mg |
|  | C08CA17 | levamlodipine | 2.5mg |
|  | C08DA01 | verapamil | 40mg |
|  | C08DB01 | diltiazem | 120mg |
|  | C08DB01 | diltiazem | 180mg |
|  | C08DB01 | diltiazem | 50mg |
|  | C08DB01 | diltiazem | 30mg |
|  | C08DB01 | diltiazem | 90mg |
|  | C09AA02 | enalapril | 10mg |
|  | C09AA02 | enalapril | 5mg |
|  | C09AA04 | perindopril | 4mg |
|  | C09AA04 | perindopril | 8mg |
|  | C09AA05 | ramipril | 2.5mg |
|  | C09AA05 | ramipril | 5mg |
|  | C09AA05 | ramipril | 10mg |
|  | C09AA06 | quinapril | 10mg |
|  | C09AA08 | cilazapril | 2.5mg |
|  | C09AA09 | fosinopril | 10mg |
|  | C09AA09 | fosinopril | 20mg |
|  | C09AA14 | temocapril | 2mg |
|  | C09AA15 | zofenopril | 7.5mg |
|  | C09AA15 | zofenopril | 15mg |
|  | C09AA15 | zofenopril | 30mg |
|  | C09AA16 | imidapril | 10mg |
|  | C09AA16 | imidapril | 5mg |
|  | C09BA01 | captopril and diuretics | 25mg+12.5mg |
|  | C09BA01 | captopril and diuretics | 50mg+25mg |
|  | C09CA01 | losartan | 50mg |
|  | C09CA01 | losartan | 100mg |
|  | C09CA03 | valsartan | 80mg |
|  | C09CA03 | valsartan | 160mg |
|  | C09CA03 | valsartan | 40mg |
|  | C09CA03 | valsartan | 320mg |
|  | C09CA04 | irbesartan | 150mg |
|  | C09CA04 | irbesartan | 300mg |
|  | C09CA06 | candesartan | 8mg |
|  | C09CA06 | candesartan | 16mg |
|  | C09CA06 | candesartan | 32mg |
|  | C09CA07 | telmisartan | 40mg |
|  | C09CA07 | telmisartan | 80mg |
|  | C09CA08 | olmesartan medoxomil | 20mg |
|  | C09CA08 | olmesartan medoxomil | 10mg |
|  | C09CA08 | olmesartan medoxomil | 40mg |
|  | C09DA01 | losartan and diuretics | 12.5mg+50mg |
|  | C09DA01 | losartan and diuretics | 25mg+100mg |
|  | C09DA01 | losartan and diuretics | 12.5mg+100mg |
|  | C09DA03 | valsartan and diuretics | 12.5mg+80mg |
|  | C09DA03 | valsartan and diuretics | 12.5mg+160mg |
|  | C09DA04 | irbesartan and diuretics | 12.5mg+150mg |
|  | C09DA04 | irbesartan and diuretics | 12.5mg+300mg |
|  | C09DA06 | candesartan and diuretics | 16mg+12.5mg |
|  | C09DA07 | telmisartan and diuretics | 12.5mg+40mg |
|  | C09DA07 | telmisartan and diuretics | 12.5mg+80mg |
|  | C09DA07 | telmisartan and diuretics | 25mg+80mg |
|  | C09DA08 | olmesartan medoxomil and diuretics | 12.5mg+20mg |
|  | C09DB01 | valsartan and amlodipine | 5mg+160mg |
|  | C09DB01 | valsartan and amlodipine | 5mg+80mg |
|  | C09DB01 | valsartan and amlodipine | 10mg+160mg |
|  | C09DB01 | valsartan and amlodipine | 5mg+160mg |
|  | C09DB02 | olmesartan medoxomil and amlodipine | 5mg+40mg |
|  | C09DB02 | olmesartan medoxomil and amlodipine | 5mg+20mg |
|  | C09DB02 | olmesartan medoxomil and amlodipine | 5mg+40mg |
|  | C09DB02 | olmesartan medoxomil and amlodipine | 10mg+40mg |
|  | C09DB02 | olmesartan medoxomil and amlodipine | 5mg+40mg |
|  | C09DB04 | telmisartan and amlodipine | 5mg+80mg |
|  | C09DB04 | telmisartan and amlodipine | 5mg+40mg |
|  | C09DB04 | telmisartan and amlodipine | 10mg+40mg |
|  | C09DB04 | telmisartan and amlodipine | 10mg+80mg |
|  | C09DB07 | candesartan and amlodipine | 5mg+8mg |
|  | C09DB07 | candesartan and amlodipine | 5mg+16mg |
|  | C09DB07 | candesartan and amlodipine | 10mg+16mg |
|  | C09DX03 | olmesartan medoxomil, amlodipine and hydrochlorothiazide | 5mg+12.5mg+40mg |
|  | C09DX03 | olmesartan medoxomil, amlodipine and hydrochlorothiazide | 5mg+12.5mg+20mg |
|  | C09DX03 | olmesartan medoxomil, amlodipine and hydrochlorothiazide | 10mg+12.5mg+40mg |
| Antibiotics | J01AA02 | doxycycline | 100mg |
|  | J01AA02 | doxycycline | 100mg |
|  | J01AA02 | doxycycline | 100mg |
|  | J01AA08 | minocycline | 50mg |
|  | J01AA12 | tigecycline | 50mg |
|  | J01CA01 | ampicillin | 500mg |
|  | J01CA04 | amoxicillin | 250mg |
|  | J01CA04 | amoxicillin | 500mg |
|  | J01CA04 | amoxicillin | 1g |
|  | J01CA04 | amoxicillin | 500mg |
|  | J01CA12 | piperacillin | 2g |
|  | J01CA12 | piperacillin | 4g |
|  | J01CE01 | benzylpenicillin | 5mg |
|  | J01CF06 | nafcillin | 1g |
|  | J01CR01 | ampicillin and beta-lactamase inhibitor | 500mg+250mg |
|  | J01CR01 | ampicillin and beta-lactamase inhibitor | 1g+500mg |
|  | J01CR02 | amoxicillin and beta-lactamase inhibitor | 125mg+62.5mg |
|  | J01CR02 | amoxicillin and beta-lactamase inhibitor | 250mg+125mg |
|  | J01CR02 | amoxicillin and beta-lactamase inhibitor | 500mg+125mg |
|  | J01CR02 | amoxicillin and beta-lactamase inhibitor | 500mg+100mg |
|  | J01CR02 | amoxicillin and beta-lactamase inhibitor | 1g+200mg |
|  | J01CR02 | amoxicillin and beta-lactamase inhibitor | 250mg+250mg |
|  | J01CR02 | amoxicillin and beta-lactamase inhibitor | 875mg+125mg |
|  | J01CR02 | amoxicillin and beta-lactamase inhibitor | 438mg+563mg+62.5mg |
|  | J01CR02 | amoxicillin and beta-lactamase inhibitor | 125mg/dose+31.3mg/dose |
|  | J01CR04 | sultamicillin | 375mg |
|  | J01CR05 | piperacillin and beta-lactamase inhibitor | 2g+250mg |
|  | J01CR05 | piperacillin and beta-lactamase inhibitor | 4g+500mg |
|  | J01DB01 | cefalexin | 500mg |
|  | J01DB01 | cefalexin | 500mg |
|  | J01DB01 | cefalexin | 500mg |
|  | J01DB03 | cefalotin | 1g |
|  | J01DB04 | cefazolin | 1g |
|  | J01DB04 | cefazolin | 2g |
|  | J01DB04 | cefazolin | 500mg |
|  | J01DB05 | cefadroxil | 250mg |
|  | J01DB05 | cefadroxil | 500mg |
|  | J01DB09 | cefradine | 250mg |
|  | J01DB09 | cefradine | 1g |
|  | J01DB09 | cefradine | 500mg |
|  | J01DB09 | cefradine | 500mg |
|  | J01DC01 | cefoxitin | 1g |
|  | J01DC02 | cefuroxime | 250mg |
|  | J01DC02 | cefuroxime | 1.5g |
|  | J01DC02 | cefuroxime | 250mg |
|  | J01DC02 | cefuroxime | 750mg |
|  | J01DC04 | cefaclor | 250mg |
|  | J01DC04 | cefaclor | 375mg |
|  | J01DC05 | cefotetan | 1g |
|  | J01DC07 | cefotiam | 1g |
|  | J01DC07 | cefotiam | 500mg |
|  | J01DC09 | cefmetazole | 1g |
|  | J01DC09 | cefmetazole | 500mg |
|  | J01DC09 | cefmetazole | 2g |
|  | J01DC10 | cefprozil | 250mg |
|  | J01DD01 | cefotaxime | 1g |
|  | J01DD01 | cefotaxime | 2g |
|  | J01DD01 | cefotaxime | 500mg |
|  | J01DD02 | ceftazidime | 1g |
|  | J01DD02 | ceftazidime | 500mg |
|  | J01DD02 | ceftazidime | 2g |
|  | J01DD04 | ceftriaxone | 1g |
|  | J01DD04 | ceftriaxone | 250mg |
|  | J01DD04 | ceftriaxone | 2g |
|  | J01DD04 | ceftriaxone | 500mg |
|  | J01DD13 | cefpodoxime | 100mg |
|  | J01DD15 | cefdinir | 100mg |
|  | J01DD17 | cefcapene | 75mg |
|  | J01DD17 | cefcapene | 100mg |
|  | J01DD62 | cefoperazone and beta-lactamase inhibitor | 500mg+500mg |
|  | J01DE01 | cefepime | 1g |
|  | J01DE01 | cefepime | 500mg |
|  | J01DE01 | cefepime | 1g |
|  | J01DE02 | cefpirome | 1g |
|  | J01DH02 | meropenem | 500mg |
|  | J01DH02 | meropenem | 1g |
|  | J01DH02 | meropenem | 2g |
|  | J01DH04 | doripenem | 250mg |
|  | J01DH51 | imipenem and cilastatin | 250mg+250mg |
|  | J01DH51 | imipenem and cilastatin | 500mg+500mg |
|  | J01EE01 | sulfamethoxazole and trimethoprim | 400mg+80mg |
|  | J01EE01 | sulfamethoxazole and trimethoprim | 400mg/dose+80mg/dose |
|  | J01FA06 | roxithromycin | 150mg |
|  | J01FA06 | roxithromycin | 50mg |
|  | J01FA09 | clarithromycin | 250mg |
|  | J01FA09 | clarithromycin | 500mg |
|  | J01FA10 | azithromycin | 250mg |
|  | J01FA10 | azithromycin | 500mg/dose |
|  | J01FF01 | clindamycin | 150mg |
|  | J01FF01 | clindamycin | 300mg |
|  | J01FF01 | clindamycin | 600mg |
|  | J01MA01 | ofloxacin | 100mg |
|  | J01MA01 | ofloxacin | 200mg |
|  | J01MA02 | ciprofloxacin | 250mg |
|  | J01MA02 | ciprofloxacin | 500mg |
|  | J01MA06 | norfloxacin | 200mg |
|  | J01MA06 | norfloxacin | 400mg |
|  | J01MA12 | levofloxacin | 100mg |
|  | J01MA12 | levofloxacin | 250mg |
|  | J01MA12 | levofloxacin | 500mg |
|  | J01MA12 | levofloxacin | 750mg |
|  | J01MA14 | moxifloxacin | 1.6mg/1ml |
|  | J01MA22 | tosufloxacin | 150mg |
|  | J01XA01 | vancomycin | 1g |
|  | J01XA01 | vancomycin | 250mg |
|  | J01XA01 | vancomycin | 500mg |
|  | J01XA02 | teicoplanin | 200mg |
|  | J01XA02 | teicoplanin | 400mg |
|  | J01XB01 | colistin | 150mg |
|  | J01XX08 | linezolid | 600mg |
|  | J01XX08 | linezolid | 2mg/1ml |
|  | J01XX09 | daptomycin | 500mg |
|  | J01XX09 | daptomycin | 350mg |
