# Supplementary material for: Comparison of generic drug prices in Korea and eight high-income countries across four therapeutic classes
Source: PLoS One. 2025 Mar 11;20(3):e0319674. doi: 10.1371/journal.pone.0319674 (PMC11896052; doi:10.1371/journal.pone.0319674)
Supplement: Attachment — Submitted filename: Response to reveiwers.docx [file pone.0319674.s003.docx]

Reviewer #1: Dear Author,

I think this article covers a very interesting topic related to generic drug prices in South Korea. However, 🡪 I would like to make a few comments about your manuscript. Follows are the details.
Thank you for your valuable comments, which help clarify some statements and correct mistakes.

1. Your research is to compare the price of a generic drug in South Korea with eight other countries (Base country: Korea). I recommend that you revise the title to reflect it.
🡪 We revised the title to “Comparison of Generic Drug Prices in Korea and Eight High-Income Countries Across Four Therapeutic Classes."

2. Explain in more detail the rationale for choosing 4 therapeutic classes and 8 comparison countries for price comparison of generic drugs.

🡪 We added additional detail for selecting drug classes; These drug classes were selected because they are considered to have high consumption levels, based on data from an international repository. (lines 101-102)

🡪 We added additional detail for selecting the eight countries for comparison; Furthermore, the authority announced a plan for a new initiative to reduce generic prices by comparing those in Korea with prices in eight other countries. (lines 110-111)

3. You selected eight countries as comparison countries for generic drug prices, which are considered as reference when determining prices in Korea. Does Korea consider the prices of the eight countries as a reference when determining the price of generic drugs?

🡪 No, they are reference countries for determining new drugs. This was clarified in line 109.

4. Despite the lower prices of generic drugs in Korea in some therapeutic classes, can we say that overall the prices of generic drugs in Korea are higher?
- I recommend that you revise the sentences in the Discussion and Conclusion sections related to this issue or provide sufficient rational.

🡪 No. It was an error to generalize the finding to overall generic prices. We revised the concerned part to “Generic drug prices for lipid-lowering and antihypertensive drugs in South Korea are higher compared to other high-income countries.” (lines 252-253)

We also added more detail (lines 261-271) in Discussion and revised the conclusion (lines 284-287).

5. Consider modifying the Table 2,3,4,5 to include both USD base and PPP USD base as shown in Table 1.

🡪 We added USD-based indices to Tables 2, 3, 4, and 5 as suggested.

6. The price indices in Figure 1 PPP USD based?

🡪 Yes. This is added as a note to Figure 1. (page 11)

7. Please make sure that manuscript was written according to the journal's guidelines as a whole. In particular, check the following:
• Please check that all abbreviations present are commonly used and all are defined in their first instance in the manuscript.
• Please check that all abbreviations present in Table/Figure legends. These should also be redefined in the legends if the abbreviation is used.
• Please make sure that all figures, tables and boxes are clearly and correctly titled, described and cited in the text.
• Please add additional detail to figure legends where relevant to ensure all figures are adequately described and appropriate context is given.

🡪 We checked the manuscript to confirm that it is written according to the guidelines and revised it when needed.

Reviewer #2:

The discussion should take into account and elaborate on the impact of drug prices, the locations of drug production, and the risk of shortages. Indeed, it is known (and observed in this work) that drug prices in France are often very low, but it also appears that supply disruptions in France are very frequent. The proximity of South Korea to Asian production lines could allow the country to prevent such problems, but this needs to be discussed further.

🡪 Thank you for your comment. We agree that the proximity of South Korea to Asian production lines could allow the country to prevent shortage problems. However, we could not find any empirical evidence confirming the link. In response to your comment, we added: Manufacturing disruptions have been considered one of the key factors contributing to drug shortages worldwide. (lines 244-245)
